# Characterization and Identification of a novel chromosome-encoded metallo-β-lactamase WUS-1 in *Myroides albus* P34

**DOI:** 10.3389/fmicb.2022.1059997

**Published:** 2022-12-01

**Authors:** Shuang Liu, Lei Zhang, Chunlin Feng, Jin Zhu, Anqi Li, Jingxuan Zhao, Yuan Zhang, Mengdi Gao, Weina Shi, Qiaoling Li, Xueya Zhang, Hailin Zhang, Teng Xu, Junwan Lu, Qiyu Bao

**Affiliations:** ^1^The Second Affiliated Hospital and Yuying Children’s Hospital, Wenzhou Medical University, Wenzhou, China; ^2^Key Laboratory of Medical Genetics of Zhejiang Province, Key Laboratory of Laboratory Medicine, Ministry of Education, China, School of Laboratory Medicine and Life Sciences, Wenzhou Medical University, Wenzhou, China; ^3^Department of Laboratory Medicine, Quzhou People's Hospital, Quzhou, China; ^4^Institute of Translational Medicine, Baotou Central Hospital, Baotou, China; ^5^Medical Molecular Biology Laboratory, School of Medicine, Jinhua Polytechnic, Jinhua, China

**Keywords:** metallo-β-lactamase, *Myroides albus*, *bla*_WUS-1_, kinetic analysis, antimicrobial resistance

## Abstract

In this study, we identified and characterized a novel chromosomally-encoded class B metallo-β-lactamase (MBL) gene designated *bla*_WUS-1_ in a carbapenem-resistant isolate *Myroides albus* P34 isolated from sewage discharged from an animal farm. Comparative analysis of the deduced amino acid sequence revealed that WUS-1 shares the highest amino acid similarities with the function-characterized MBLs MUS-1 (AAN63647.1; 70.73%) and TUS-1 (AAN63648.1; 70.32%). The recombinant carrying *bla*_WUS-1_ exhibited increased MICs levels against a number of β-lactam antimicrobials such as carbenicillin, ampicillin and imipenem, and β-lactamase inhibitors (clavulanic acid and tazobactam). The metallo-β-lactamase WUS-1 could also hydrolyze these antimicrobials and the hydrolytic activities could be inhibited by EDTA. Genetic context analysis of *bla*_WUS-1_ revealed that no mobile genetic element was found in its surrounding region. The plasmid pMA84474 of *Myroides albus* P34 harbored 6 resistance genes (*bla*_OXA-347_, *aadS*, *bla*_MYO-1_, *ereD*, *sul2* and *ermF*) within an approximately 17 kb multidrug resistance (MDR) region. These genes, however, were all related to mobile genetic elements.

## Introduction

Bacteria of the genus *Myroides* are peculiar opportunistic and extensively antibiotic-resistant pathogens, which originally belonged to the genus *Flavobacterium* (Bergey et al., 1923). They are well known for their resistance to antibiotics that are generally used in the treatment of infections caused by gram-negative bacteria (Holmes et al., 1979). *Myroides* strains have been gradually implicated as important nosocomial pathogens, as infections caused by *Myroides* spp. have been increasingly reported. The earliest recorded isolation of the species, previously named *Flavobacterium breve,* was from the human intestine by Stutzer (Bergey et al., 1923). In 1984, Holmes et al. had suggested that *F. odoratum* should be classified in a separate genus on the basis of its unique phenotypic features (Holmes et al., 1984). Differing from most *Flavobacterium* strains, being nonsaccharolytic and failing to produce indole, researchers suggested that *F. odoratum* should be excluded from the emended genus *Flavobacterium* based on its unique genotypic, chemotaxonomic, and phenotypic data (Bernardet et al., 1996). Vancanneyt et al. conducted a general polyphasic taxonomy analysis and placed *F. odoratum* into a separate new genus, *Myroides* (Vancanneyt et al., 1996). The first two species to be delineated were *Myroides odoratus* comb. nov. and *Myroides odoratimimus* sp. nov.

Members of the genus *Myroides* are nonmotile, nonfermentative, obligately aerobic, gram-negative rods that produce a characteristic fruity odor and yellow pigment. Although the strains are widely distributed in environmental ecosystems, such as oil, and fresh and marine waters (Maneerat et al., 2006; Yoon et al., 2006), they have also been previously reported in insect guts (Spiteller et al., 2000). The species has also been reported to be frequently isolated from human urine, wound discharge, sputum, and blood (Ahamed et al., 2018; Cruz-Choappa et al., 2021; O'Neal et al., 2022), but it is not normally a part of the human microflora (Shewan and McMeekin, 1983). Definite cases of human infection caused by this organism are rather rare and generally occur in immunocompromised individuals. However, while infections are sometimes life-threatening, they rarely affect immunocompetent hosts (Maraki et al., 2012). *Myroides* organisms have been considered to be responsible for cases of pneumonia, endocarditis, urinary tract infections, ventriculitis, bacteremia, and soft-tissue infections (Macfarlane et al., 1985; Ferrer et al., 1995; Green et al., 2001; Crum-Cianflone et al., 2014; Deepa et al., 2014; Faraz et al., 2022). In addition, nosocomial outbreaks such as urinary tract infections and other central venous catheter-associated bloodstream infections caused by *Myroides* spp. due to contaminated water have been reported (Douce et al., 2008; Ktari et al., 2012; Kutlu et al., 2020). Infections caused by *Myroides* spp. are not common, but antibiotic therapy is troublesome due to their resistance to virtually all β-lactam antimicrobials, including carbapenems. Reference reports indicated that *Myroides* strains exhibit variable susceptibility to β-lactam antibiotics (Holmes et al., 1979), with a decreased susceptibility to cephalosporins and carbapenems (Mammeri et al., 2002), and MBLs are found to play a significant role. MBLs differ structurally from the other β-lactamases based on their requirement for active-site zinc ions for activity, and their catalysis proceeds by directly attacking hydroxide ions (Wang et al., 1999; Crowder et al., 2006). MBLs show little or no susceptibility to mechanism-based inhibitors of serine enzymes, such as clavulanic acid, sulbactam or tazobactam, but can be inactivated by metal chelators such as EDTA (Drawz and Bonomo, 2010). MBLs were discovered approximately 50 years ago and were initially identified as clinically irrelevant enzymes that were chromosomally encoded in occasional bacilli such as *Bacillus* and *Aeromonas* (Lim et al., 1988; Walsh et al., 1994). They were not taken seriously, resulting in far less study compared to other β-lactamases, until the metalloenzyme IMP-1 was discovered in Japan in the 1990s and the encoding gene was found to be located on an integron. Horizontal transfer is mediated by the movement of integrons and the insertion and removal of gene boxes (Watanabe et al., 1991). At present, a variety of metalloenzymes that can be horizontally transferred have been found (De Oliveira et al., 2020; Li et al., 2022). With the widespread application of carbapenems, the potential spread of metalloenzymes among pathogenic bacteria is a frightening possibility, which emphasizes the importance of understanding their properties.

In this study, the first complete genome of a *Myroides albus* strain is presented. We characterized WUS-1 by researching its catalytic abilities and impacts on antimicrobial susceptibility. In addition, multiple sequence alignments and phylogenetic analysis were performed to investigate the role of WUS-1 in the intrinsic resistance mechanisms of this unusual opportunistic pathogen to β-lactam antibiotics. We are looking forward to providing valuable information for the study of *Myroides* drug resistance characteristics.

## Materials and methods

### Bacterial strains and plasmids

*Myroides albus* P34 was isolated from sewage discharged from an animal farm in Wenzhou, Zhejiang Province, China. The sewage samples were inoculated onto the blood plate, and incubated for 24 h at 35°C. Individual colonies of different color and size were randomly selected and preliminarily confirmed by a Vitek-60 microorganism autoanalysis system (bioMe’rieux Corporate, Craponne, France). Further identification was performed by sequencing the *16S rRNA* gene and performing ANI analyses. *E. coli* DH5α and *E. coli* BL21 were used as the hosts for the resistance gene cloning and protein overexpression, respectively. The *bla*_WUS-1_ gene was first cloned into the pUCP24 plasmid, and then the pCold I vector was used to induce cold shock expression of histidine-tagged WUS-1. All the strains and plasmids used in this work are listed in Table 1.

**Table 1:** Bacteria and plasmids used in this work.

Strain or plasmid	Relevant characteristic(s)	Reference or source
Strains		
P34	The wild-type strain of *Myroides albus* P34	This study
DH5α	*E. coli* DH5α was used as a host for cloning of the *bla*_WUS-1_ gene	Our laboratory collection
BL21	*E. coli* BL21 was used as a host for expression of WUS-1.	Our laboratory collection
ATCC 25922	*E. coli* ATCC 25922 was used as a quality control for antimicrobial susceptibility testing	Our laboratory collection
pUCP24-*bla*_WUS-1_/DH5α	DH5α carrying the recombinant plasmid pUCP24-*bla*_WUS-1_	This study
pCold I-*bla*_WUS-1_/BL21	BL21 carrying the recombinant plasmid pCold I-*bla*_WUS-1_	This study
Plasmid		
pUCP24	Cloning vector for the PCR products of the *bla*_WUS-1_ gene with its upstream promoter region, GEN^r^	Our laboratory collection
pCold I	Protein expression vector for the PCR products of the ORF of the *bla*_WUS-1_ gene, AMP^r^	Our laboratory collection

r Resistance; GEN, gentamicin; AMP, ampicillin.

### Antimicrobial susceptibility testing

Antimicrobial susceptibility testing was conducted using the agar dilution method following the guidelines of the Clinical and Laboratory Standards Institute (CLSI). Antimicrobials used in this study included carbenicillin, ampicillin, ticarcillin, amoxicillin-clavulanate, ampicillin-sulbactam, piperacillin-tazobactam, cefazolin, cefoxitin, ceftazidime, cefepime, aztreonam, imipenem, meropenem, florfenicol, chloramphenicol, nalidixic acid, tetracycline, streptomycin, kanamycin, netilmicin, gentamicin, and amikacin. When neither the CLSI M100 (32nd Edition, 2022) nor European Enterobacteriaceae Antimicrobial Susceptibility Test Committee (European Committee on Antimicrobial Susceptibility Testing. [EUCAST], 2021) provided exact breakpoints for *Myroides* spp., the minimum inhibitory concentration (MIC) values were interpreted following the criteria for non-Enterobacteriaceae bacteria (such as *A. faecalis*, *Elizabethkingia* or *Chryseobacterium*; CLSI, 32nd Edition, 2022). *E. coli* ATCC 25922 was used as a quality control in each MIC test.

### Whole-genome sequencing and functional annotation of the genome sequence of *Myroides albus* P34

Genomic DNA extraction of *Myroides albus* P34 was performed using the AxyPrep Bacterial Genomic DNA Miniprep kit (Axygen Scientific, Union City, CA, United States) and was sequenced on both the Illumina HiSeq 2500 and PacBio RS II platforms by Shanghai Personal Biotechnology Co., Ltd. (Shanghai, China). The long PacBio reads were initially assembled by Canu (Koren et al., 2017), and then the sequence generated by the Illumina HiSeq 2500 platform was aligned to the primary assembly to correct for possible assembly errors using BWA v0.7.12 (Li and Durbin, 2009) and Pilon (Walker et al., 2014). Potential open reading frames (ORFs) were predicted using Prokka v1.14.6 (Seemann, 2014). Further functional annotation of predicted proteins was performed using DIAMOND (Buchfink et al., 2015) and searching against the NCBI nonredundant protein database with an e-value threshold of 1e-5. Resistance genes were annotated using the Resistance Gene Identifier (RGI) of the Comprehensive Antibiotic Resistance Database (CARD; McArthur et al., 2013). The ANI was calculated using FastANI v1.31 (Jain et al., 2018). Mobile genetic elements (MGEs) were detected using ISFinder (Siguier et al., 2006) and INTEGRALL (Moura et al., 2009). CGView Server (Petkau et al., 2010) was used to depict the circular map of the plasmid. Multiple amino acid sequence alignments and neighbor-joining phylogenetic tree constructions for WUS-1 and other class B1 MBLs sharing the closest amino acid sequence similarities to WUS-1 were performed using MAFFT (Katoh and Standley, 2013) and MEGAX (Kumar et al., 2018), respectively. Linear maps of genomic structure and structural comparisons of multiple genomes were portrayed using genoPlotR (Guy et al., 2010). The promoter region of *bla*_WUS-1_ was predicted by BPROM.^1^ The molecular weight and pI value of WUS-1 were predicted using the Expasy ProtParam Tool,^2^ and the putative signal peptide cleavage site was predicted by SignalP 5.0 (Almagro Armenteros et al., 2019).

### Phenotypic detection of carbapenemases

The strain *Myroides albus* P34 was screened for carbapenemase production by the modified Hodge test, in the meantime, the mCIM and eCIM tests following the CLSI M100 (32nd Edition, 2022) specifications were performed for carbapenemase phenotypic detection.

### Cloning of the *bla*_WUS-1_ gene and expression and purification of WUS-1

Amplification of the chromosomal *bla*_WUS-1_ gene with its promoter region was performed using primers (Table 2) that contained *Bam*HI and *Hind*III restriction endonuclease adapters at the 5′ ends. The PCR product was eluted from the agarose gel, digested with the restriction endonucleases *Bam*HI and *Hind*III (Takara Bio, Inc., Dalian, China), and ligated into the pUCP24 vector using the T4 DNA ligase cloning kit (Takara Bio, Inc., Dalian, China). Recombinant plasmids were introduced into *E. coli* DH5α by the calcium chloride method. A single transformant was selected on a Luria-Bertani (LB) agar plate containing 40 mg/L gentamicin, and the cloned DNA fragment of the recombinant plasmid was confirmed by both PCR and PCR product Sanger sequencing (Shanghai Sunny Biotechnology Co., Ltd., Shanghai, China). Furthermore, antimicrobial susceptibility testing for the transformants was carried out as described above.

**Table 2:** Cloning primers for the *bla*_WUS-1_ gene.

Primer^a^	Sequence (5′–3′)^b^	Restriction endonuclease	Vector	Annealing temperature (°C)	Amplicon size (bp)
*orf*-*bla*_WUS-1_-F	CGCGGATCCCTGGTGCCGCGCGGCAGCTCCGAAAGACTAAAGATCGAAAAG	*Bam*HI + Thrombin	pCold I	54	681
*orf-bla*_WUS-1_ *-R*	CCCAAGCTTGGGTTAATTGTTTTTATTTAAATTATCTCGATGGTATTCC	*Hin*dIII	pCold I	54	681
*pro*-*bla*_WUS-1_-F	CGCGGATCCGCGAGATAACGACTGAGTTATTTGATCAA	*Bam*HI	pUCP24	53	1,008
*pro*-*bla*_WUS*-*_-R	CCCAAGCTTGGGTTAATTGTTTTTATTTAAATTATCTCGATGGT	*Hin*dIII	pUCP24	53	1,008

a Primers with “orf” were used to clone the ORF of the bla_WUS-1_ gene, and primers with “pro” were used to clone the bla_WUS-1_ gene with its promoter region.

b The underlined sequences represent the restriction endonuclease sites and their protective bases.

The PCR amplification product without a signal peptide but containing a thrombin cleavage site and a pair of flanking restriction endonuclease adapters (a *Bam*HI site and a thrombin cleavage site were included in the orf-*bla*_WUS-1_-F forward primer, and a *Hind*III site was included in the orf-*bla*_WUS-1_-R reverse primer; shown in Table 2) was digested with *Bam*HI and *Hind*III and then cloned into plasmid pCold I (digested with the same restriction enzymes). The recombinant plasmids were subsequently transformed into *E. coli* BL21 by the calcium chloride method, and the transformants were selected on LB agar plates containing 100 mg/L ampicillin. The transformant was further screened by PCR and sequencing and cultured in LB liquid medium with 100 mg/L ampicillin at 37°C overnight. Then, the overnight culture was diluted in LB liquid medium at a ratio of 1:100 and further cultured until it reached an OD_600_ between 0.6 and 0.8. Protein expression was induced by incubating for 12 h at 16°C with isopropyl-β-d-thiogalactoside (IPTG; Sigma Chemicals Co., St. Louis, MO, United States) at a final concentration of 1 mM. Cells were harvested and resuspended and then disrupted sonically. The supernatant containing the β-lactamase was purified by a BeyoGold His-tag Purification Resin kit (Beyotime, Shanghai, China) according to the manufacturer’s instructions. Then, the His-tag was removed by incubating with thrombin (GenScript, Nanjing, China) for 12 h at 16°C. Finally, protein purification kits were applied to remove the free His-tag from the incubated protein. The purity and relative molecular mass of enzymes were checked using SDS–PAGE analysis, and the protein concentration was determined spectrophotometrically with a BCA Protein Assay Kit (Beyotime, Shanghai, China).

### Determination of kinetic parameters

The enzymatic activities of purified WUS-1 were measured by a continuous spectrophotometric assay with a SpectraMax multifunctional microplate reader (M5, Molecular Devices, America). The rates of hydrolysis were calculated by monitoring the absorbance drop in a 200 μL reaction mixture containing 10 mM phosphate buffer (pH 7.4) and 50 μM ZnCl_2_ at 30°C (Mammeri et al., 2002). Consequent data were analyzed by GraphPad Prism 9 software (GraphPad Software, San Jose, CA, United States) using nonlinear regression of initial reaction rates with the Michaelis–Menten equation to calculate the steady-state kinetic parameters (*kcat* and *Km*). The enzyme kinetic parameters were calculated following the formula.


v=(Vmax[S])/(Km+[S])

Where v is velocity, Vmax is maximum velocity when all the enzymes are complexed to the substrate, [S] is substrate concentration and *Km* is Michaelis–Menten constant. The wavelengths are shown in Supplementary Table S1. To investigate the effects of inhibitors, the enzyme was preincubated with various concentrations of EDTA for 5 min at 30°C before the addition of substrate (100 μM imipenem; Mammeri et al., 2002). Fifty percent inhibitory concentrations (IC_50_) were required to reduce the hydrolysis of 100 μM imipenem by 50%, and the results were determined by nonlinear regression in Prism software (Chen et al., 2019).

## Results

### Genome characteristics, carbapenemase phenotype and resistance profile of *Myroides albus* P34

The colony of *Myroides albus* P34 is medium sized with fruity odor and yellow pigment. It is positive for oxidase, urease and phosphatase, but negtative for catabolizing glucose, lactose, maltose, mannitol, sucrose, aesculin, or indole. It was preliminary identified as *Myroides* spp. The *16S rRNA* gene of isolate P34 had the closest relationship with that of *Myroides albus* BIT-d1 (MK734183.1) with 100% coverage and 100% identity. ANI analysis showed that it shared the highest identity (99.38%) with the *Myroides albus* strain BIT-d1 Scaffold1 (NZ_WMJX01000001). Therefore, this isolate was designated *Myroides albus* P34. The complete genome of *Myroides albus* P34 consisted of a 3,701,500 bp chromosome and one circular plasmid of approximately 84,474 bp in length. The chromosome and the plasmid encoded 3,218 and 95 coding sequences (CDSs) with an average GC content of 34.18 and 31.07%, respectively (Supplementary Table S2). Sequence homology analysis showed that the plasmid shared the highest similarity to the plasmid p63039 of *Myroides odoratimimus* PR63039 (CP013691.1) at 91% coverage and 99.71% identity. Similar to most strains of the genus *Myroides*, *in vitro* susceptibility tests showed that *Myroides albus* P34 exhibited resistance to all antimicrobials tested except for chloramphenicol, with especially high MIC levels for carbapenems (imipenem >512 mg/L and meropenem >512 mg/L, Table 3). The modified Hodge test and the mCIM and eCIM tests (zone diameter of 6 mm for the mCIM test and 20 mm for the eCIM test with a zone diameter difference of 14 mm) were all positive, suggesting metallo-β-lactamase production. To investigate whether any novel MBL gene was encoded in the *Myroides albus* P34 genome, we checked the whole genome annotation result and found that in addition to one plasmid-encoded, function-characterized MBL gene (*bla*_MYO-1_), the P34 genome also harbored six chromosomally encoded hypothetical MBL genes. We analyzed the sequence structures of the predicted MBL genes and found that one (finally designated *bla*_WUS-1_) contained conserved motifs of an Ambler class B1 β-lactamase. We then cloned the gene and determined its function.

**Table 3:** MICs of 22 antimicrobials for P34, the recombinant carrying *bla*_WUS-1_ and the control strains (mg/L).

Antibiotics	*Myroides* P34	pUCP24-*bla*_WUS-1_/DH5α	pUCP24/DH5α	DH5α	ATCC 25922
Carbenicillin	>1,024	1,024	4	4	4
Ampicillin	>1,024	64	4	2	4
Ticarcillin	>1,024	256	8	8	8
Piperacillin	256	16	4	2	4
Ticarcillin clavulanate	>1,024	256	2	4	8
Ampicillin sulbactam	1,024	32	2	2	2
Piperacillin tazobactam	128	32	2	2	2
Cefazolin	>1,024	2	2	2	2
Cefoxitin	512	2	2	2	2
Ceftazidime	>128	0.5	0.25	0.25	0.25
Cefepime	>64	<0.03	<0.03	0.06	0.06
Aztreonam	128	<0.03	0.125	0.125	0.125
Imipenem	>512	1	0.125	0.06	0.125
Meropenem	>512	0.5	0.125	0.125	0.06
Florfenicol	8	-	-	4	<4
Chloramphenicol	8	-	-	8	4
Nalidixic acid	32	-	-	8	4
Tetracycline	64	-	-	2	2
Streptomycin	1,024	-	-	2	2
Kanamycin	>128	-	-	2	1
Netilmicin	>64	-	-	8	2
Gentamicin	64	-	-	<0.125	0.25
Amikacin	128	-	-	1	4

### Homologs of the novel metallo-β-lactamase WUS-1

**Figure 1:**
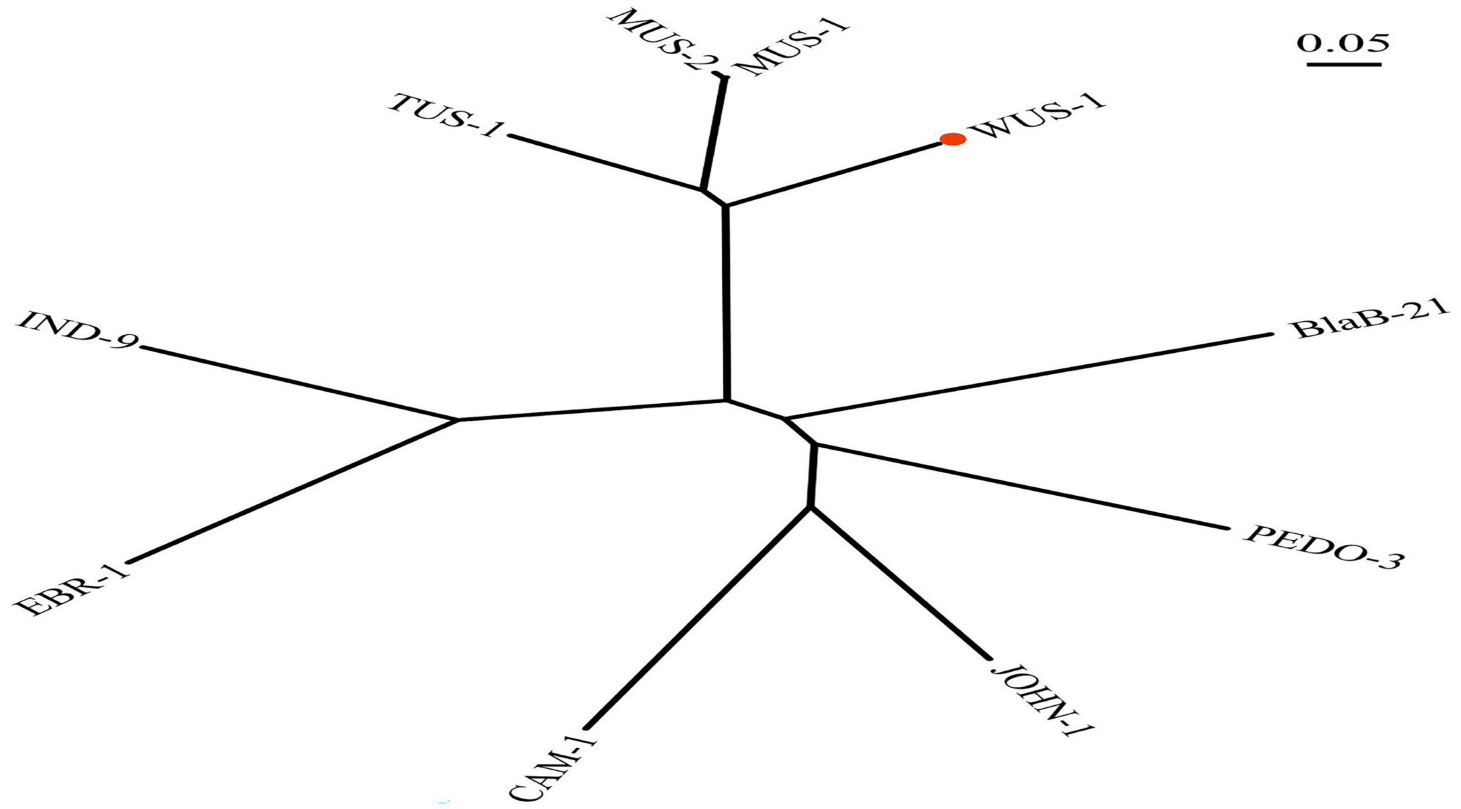
Phylogenetic tree showing the relationship of WUS-1 to other class B β-lactamases. WUS-1 from this study is marked with a red dot. The sequences and their accession numbers are as follows: MUS-1 (AAN63647.1), MUS-2 (AKN19901.1), TUS-1 (AAN63648.1), IND9 (ACZ65153.1), EBR-1 (AAN32638.1), CAM-1 (AVX51087.1), JOHN-1 (AAK38324.1), PEDO-3 (AJP77076.1), and BlaB-21 (AIL46641.1).

**Figure 2:**
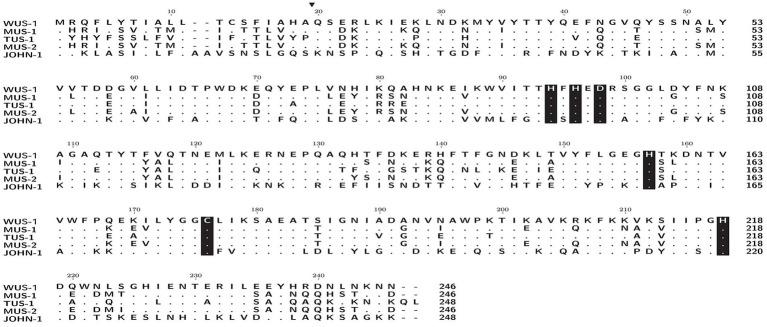
Amino acid alignment of WUS-1 with other function-characterized class B β-lactamases. Dots indicate amino acid residues identical to those of WUS-1. Amino acid residues that may be involved in Zn2+ binding are shaded in black. The predicted signal sequence cleavage site for WUS-1 is indicated by a black arrow. The sequences and their accession numbers are as follows: MUS-1 (AAN63647.1), TUS-1 (AAN63648.1), MUS-2 (AKN19901.1), and JOHN-1 (AAK38324.1).

The *bla*_WUS-1_ gene is 741 bp in length and encodes a predicted protein of 246 amino acids with a calculated molecular mass of *ca.* 28.64 kDa. The predicted cleavage site of the signal peptide is found between positions 19 and 20 (alanine and glutamine residues). After removal of the signal peptide, the enzyme is a *ca.* 26.33 kDa protein with a predicted pI value of 7.09. Nine functionally characterized protein sequences with the highest similarity (ranging from 38.36–70.73%) to WUS-1 were retrieved from the NCBI nonredundant (NR) protein database, and they were all class B1 β-lactamases, of which the β-lactamase MUS-1 (AAN63647.1) ranked the first. The phylogenetic tree for these proteins was constructed and showed that WUS-1 is most closely related to MUS-1 from *M. odoratimimus* CIP 103073 (100% coverage and 70.73% identity), TUS-1 from *M. odoratus* CIP 103105 (100% coverage and 70.32% identity) and MUS-2 from *M. odoratimimus* 35a (100% coverage and 69.92% identity; Figure 1). Alignment result of the deduced amino acid sequence of WUS-1 with the other 4 closest relatives (identities ranging from 42.33–70.73%) is shown in Figure 2. It showed that they shared two strictly conserved motifs of the subclass B1 metallo-β-lactamases that interact with the Zn^2+^ cofactor or with the water molecule located in the active site. The Zn1 binding site, also known as the 3H site, contains three histidine residues, His94, His96 and His157, while the ligands for the Zn2 or DCH site include Asp98, Cys176 and His218 (Garau et al., 2004).

**Figure 3:**
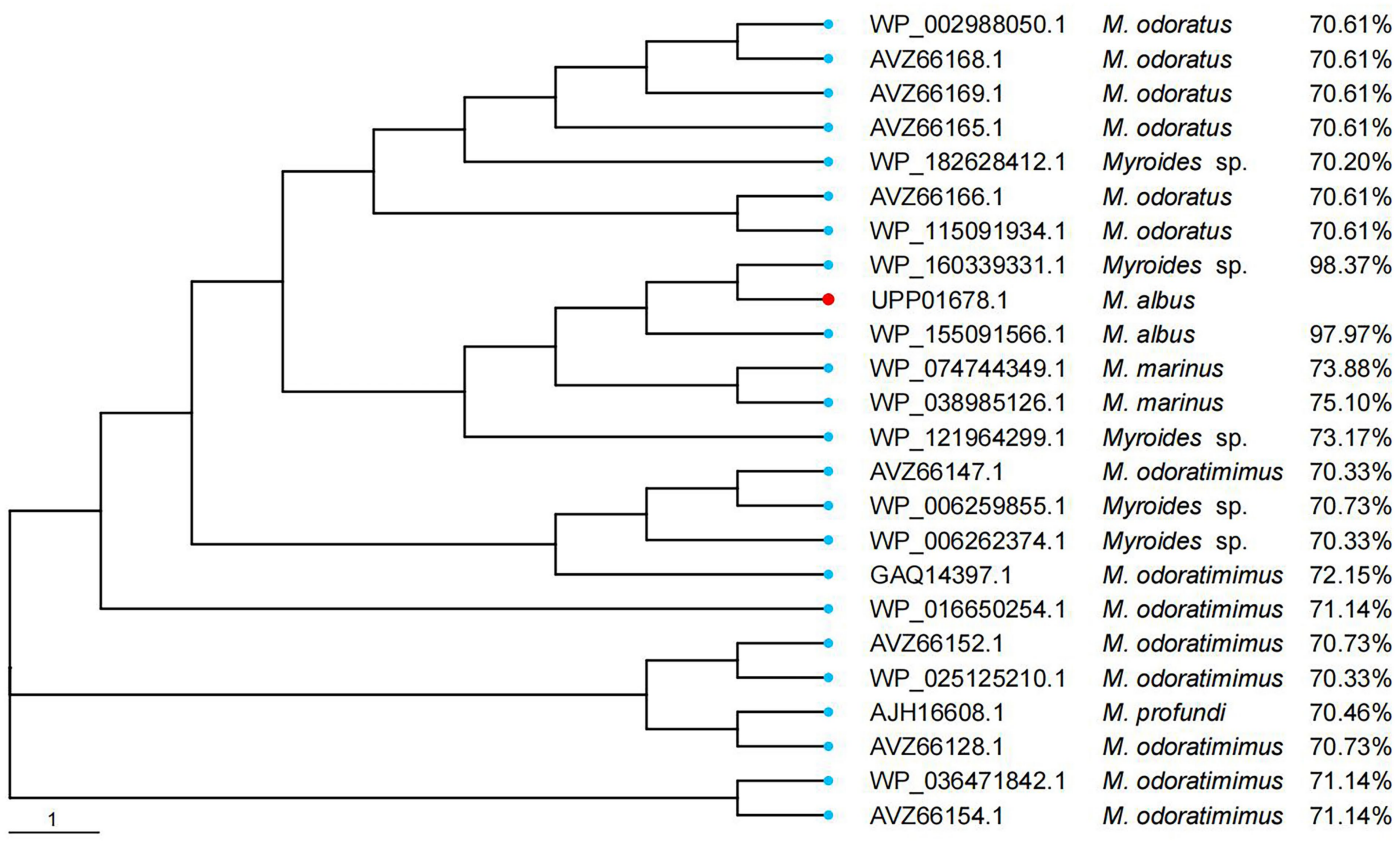
Phylogenetic analysis of WUS-1 with other putative class B β-lactamases (≥70% amino acid similarity). WUS-1 from this study is represented with a red dot.

When searching for WUS-1 homologous proteins (>70% amino acid similarity) in the NCBI NR database, 23 functionally uncharacterized sequences (percent identities between 70.2 and 98.37%) were retrieved (Figure 3), all of which were from the *Myroides* species. The closest relative of WUS-1 was a hypothetical class B enzyme from *Myroides* sp. Loew2-1 (WP_160339331.1), and they shared an identity of 98.37% with a coverage of 100%.

### Functional characteristics of the *bla*_WUS-1_ gene

The recombinant strain pUCP24-*bla*_WUS-1_/DH5α showed increased MIC levels for ampicillin (16-fold), carbenicillin (256-fold), ticarcillin (32-fold), imipenem (8-fold) and meropenem (4-fold) in comparison with the control strains (DH5α carrying the vector pUCP24), whereas no MIC level change was observed for expanded spectrum cephalosporins or aztreonam (Table 3). Clavicularulanic acid and sulbactam did not restore the activity of β-lactams. WUS-1 displayed extremely low catalytic efficiencies *(kcat/Km*) for cefazolin and cefoxitin (Table 4). In accordance with other subclass B1 β-lactamases, the catalytic efficiencies of WUS-1 for cephalosporins were lower than those of penicillins or carbapenems (Mammeri et al., 2002; Vessillier et al., 2002; Naas et al., 2003). The highest catalytic efficiency was observed with ampicillin (*kcat/Km* rate of 10.06 μM^−1^·s^−1^). The catalytic efficiency for carbenicillin (1.93 μM^−1^·s^−1^) was lower than that of carbapenems. Carbapenems behaved as good substrates, and the high *kcat/Km* ratios observed with imipenem (3.99 μM^−1^·s^−1^) and meropenem (3.24 μM^−1^·s^−1^) always resulted from very high turnover rates. Cefoxitin (0.32 μM^−1^·s^−1^) and cefazolin (0.57 μM^−1^·s^−1^) were poorly hydrolyzed by WUS-1, and aztreonam was not hydrolyzed at all. The result of the β-lactamase activity inhibition analysis, as measured by the IC_50_ (50% inhibitory concentration), demonstrated that the activity of WUS-1 was inhibited by EDTA (IC_50_ of 71.28 μM for WUS-1).

**Table 4:** Kinetic parameters of β-lactam antibiotics for the β-lactamase WUS-1.

Substrate	*K_m_* (μM)	*k_cat_* (s^−1^)	*k_cat_/K_m_* (μM^−1^·s^−1^)
Carbenicillin	581	1,119.91	1.93
Ampicillin	290.2	2,920.7	10.06
Ticarcillin	827.4	702.78	0.85
Cefoxitin	44.33	14.17	0.32
Cefazolin	59.86	33.86	0.57
Imipenem	334.8	1,335.49	3.99
Meropenem	147.4	477.45	3.24
Aztreonam	NH^a^	NH^a^	NH^a^
Ceftazidime	NH^a^	NH^a^	NH^a^
Cefepime	NH^a^	NH^a^	NH^a^

Values are the means of three independent measurements. Standard deviations were within 15%.

a NH, no detectable hydrolysis.

### Analysis of the genetic context of *bla*_WUS-1_

**Figure 4:**
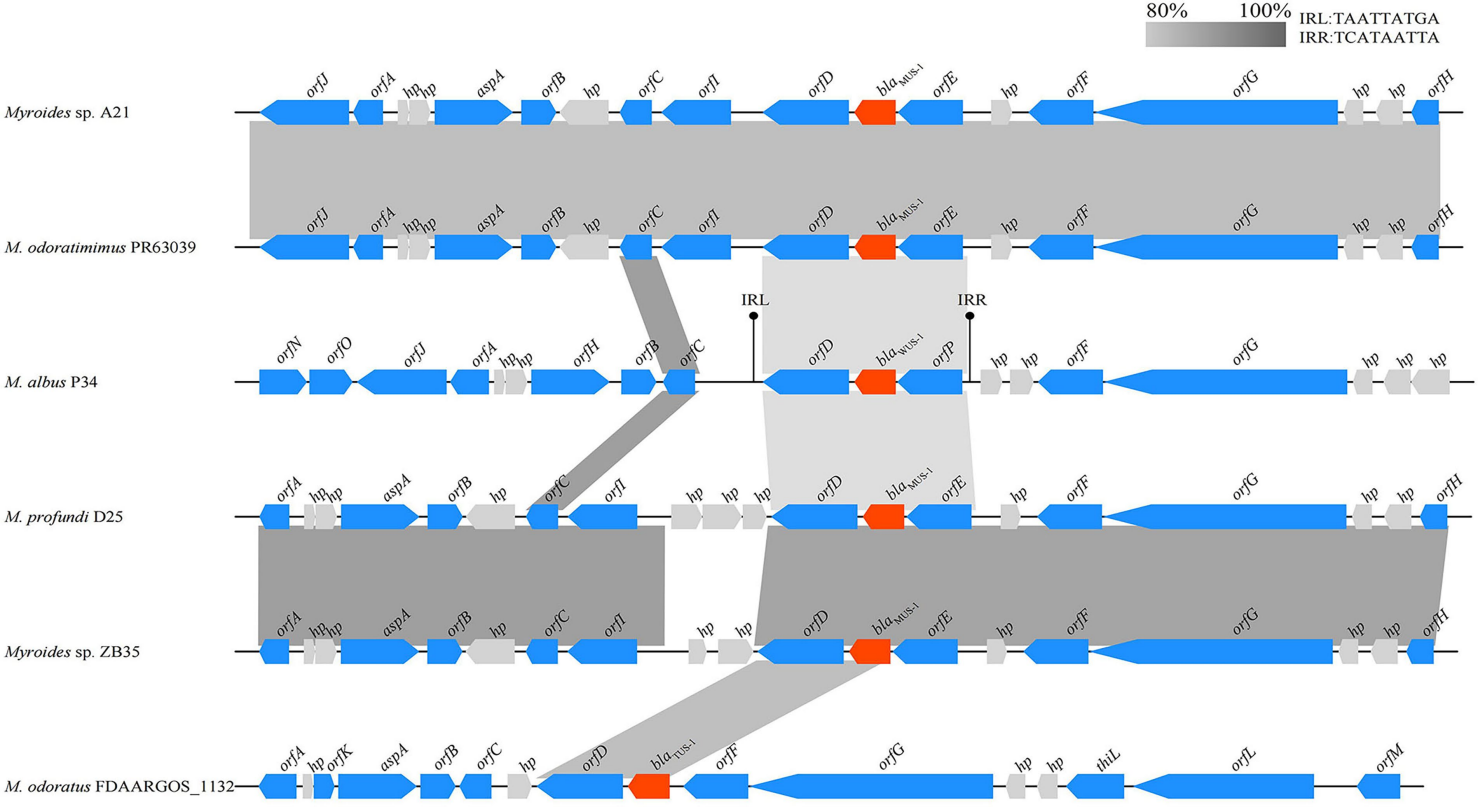
Comparison of the genetic environment of the blaWUS-1 gene with those sequences carrying its homologous genes. Genes are shown as arrows and colored based on gene function classification. Genes without functional annotation are colored in gray. Shading denotes homologous regions between them. The accession numbers are as follows: Myroides sp. A21 chromosome (CP010327.1), Myroides odoratimimus PR63039 chromosome (NZ_CP013690.1), M. profundi D25 chromosome (NZ_CP010817.1), Myroides sp. ZB35 chromosome (NZ_CP017769.1) and M. odoratus FDAARGOS_1132 chromosome (CP068107.1).

The *bla*_WUS-1_ gene was located on the chromosome of *Myroides albus* P34. To compare the genetic environment of *bla*_WUS-1_ with those of homologous genes, a comparative genomic analysis was carried out on a nucleotide sequence of approximately 21 kb in length containing the upstream and downstream flanking regions of the *bla*_WUS-1_ gene. Five chromosome segments with *bla*_WUS-1_ equivalent genes that shared the highest sequence similarities in the center were found from the NCBI database (similarities ranging between 78.89 and 81.01%), and they were all from *Myroides* spp. strains (Figure 4). The genes upstream and downstream of the *bla*_WUS-1_-related region were quite different from those sequences carrying counterpart genes. However the *orfD-bla*_WUS-1_-*orfP* locus was relatively conserved and flanked by a pair of 9 bp imperfect inverted repeats (IRs), suggesting that sequence variation might result from rearrangement mediated by the inverted repeats.

### Comparative genomics analysis of the plasmid carrying multiple resistance genes

**Figure 5:**
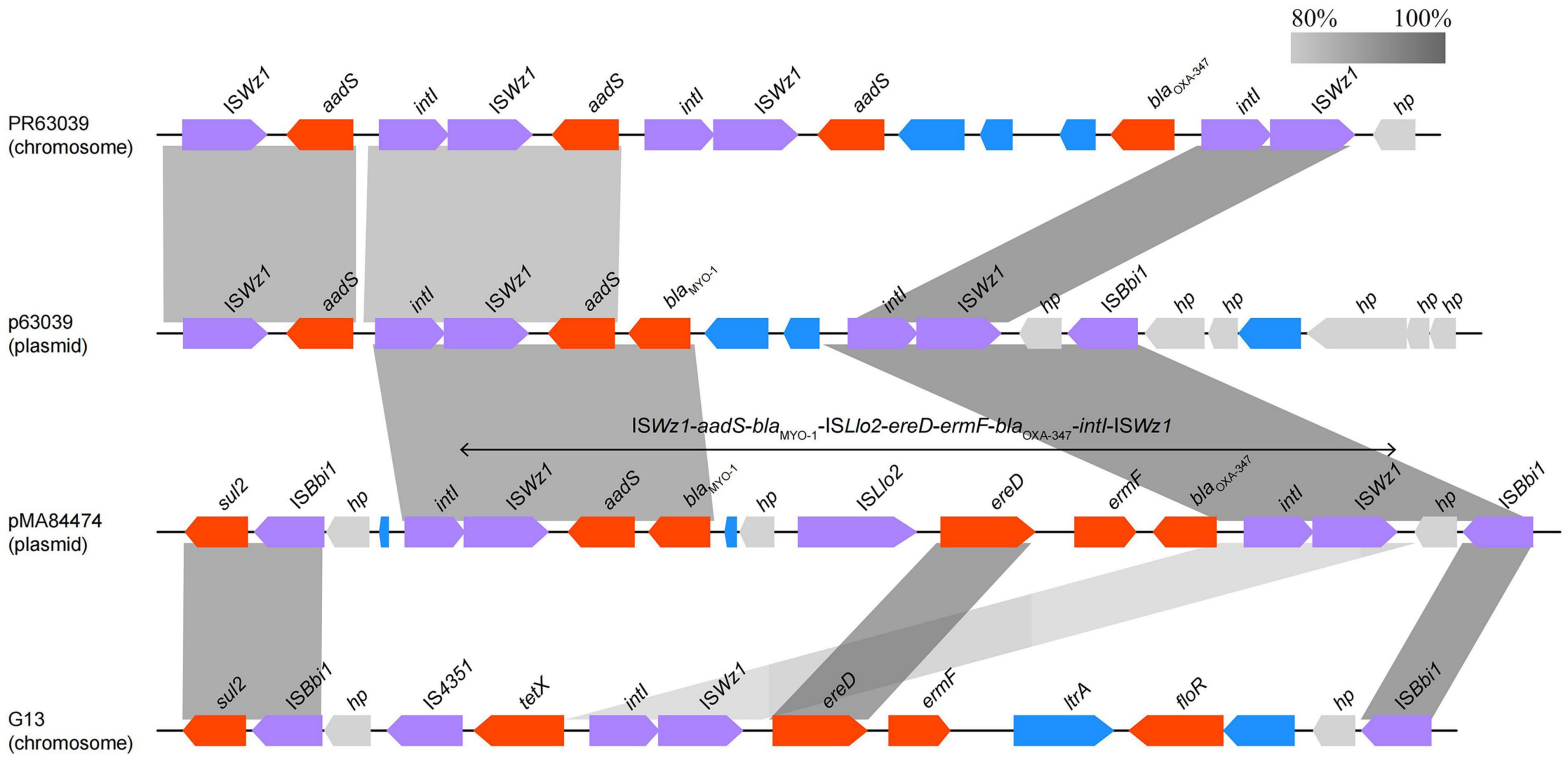
Comparison of the MDR region of pMA84474 with the related sequences. Genes are denoted by arrows and colored according to gene function classification. Shading indicates the homologous regions. The sequences and accession numbers are Myroides odoratimimus PR63039 chromosome (NZ_CP013690.1), Myroides odoratimimus p63039 plasmid (NZ_CP013691.1), and Myroides odoratimimus G13 chromosome (NZ_CP037427.1).

Analyzing the complete genome sequence of *Myroides albus* P34, we found that the isolate contained a circular plasmid designated pMA84474, which shared the highest identity with the plasmid p63039 of *Myroides odoratimimus* PR63039 (CP013691.1, 91% coverage and 99.71% identity) retrieved from the NCBI nucleotide database. The annotation result showed that there were six drug-resistance genes and a series of mobile genetic elements densely distributed in the multidrug resistance (MDR) region (20.2–37.5 kb) of pMA84474 (Figure 5), and the six resistance genes included two β-lactam resistance genes (*bla*_OXA-347_ and *bla*_MYO-1_), one aminoglycoside drug resistance gene (*aadS*), two macrolide resistance genes (*ereD* and *ermF*), and one sulfonamide resistance gene (*sul2*). Comparative genomic analysis showed that the MDR region of pMA84474 shared the highest nucleotide sequence similarity with sequences in the plasmid p63039 (CP013691.1, 59.0% coverage and 93.69% identity) and the chromosomes of *Myroides odoratimimus* PR63039 (CP013690.1, 77.0% coverage and 94.83% identity) and *Myroides odoratimimus* G13 (CP037427.1, 66.0% coverage and 99.56% identity). The five resistance genes were clustered in a mobile genetic element-related unit flanked by a pair of IS*91* family transposase IS*Wz1* sequences (IS*Wz1*-*aadS*-*bla*_MYO-1_-IS*4*-*ereD*-*ermF*-*bla*_OXA-347_-*intI*-IS*Wz1*). IS*91* is the prototype element of a family of bacterial insertion sequences and is able to perform one-ended transposition with high efficiency, which raises the possibility of IS*91*-mediated gene spreading without the need to assemble a compound transposon (Garcillan-Barcia and de la Cruz, 2002).

## Discussion

In this study, we identified a novel chromosomal class B metallo-β-lactamase gene, *bla*_WUS-1_, in the environmental *Myroides albus* isolate P34. Phylogenetic analysis showed that WUS-1 establishes a new branch in the class B β-lactamase family.

MBLs are Zn^2+^-dependent β-lactamases. They are classified as class B β-lactamases based on the Ambler molecular structure classification (Ambler, 1980) or as group 3 according to the Bush-Jacoby-Medeiros functional classification (Bush et al., 1995). MBLs can be further divided based on either structure (subclasses B1, B2, and B3; Garau et al., 2004, Frere et al., 2005, Galleni et al., 2001) or function (subgroups 3a, 3b, and 3c; Rasmussen and Bush, 1997). Sequence analysis revealed that WUS-1 contains two zinc ion binding sites and belongs to subclass B1 (Frere et al., 2005). Furthermore, WUS-1 demonstrated high catalytic efficiencies with penicillin and had *kcat/Km* values comparable to those of imipenem. Taking into consideration the biochemical criteria established by Rasmussen and Bush (Rasmussen and Bush, 1997), WUS-1 belongs to functional subgroup 3a. Most MBLs display an extremely broad substrate profile and are able to inactivate most clinically useful β-lactam antibiotics (Crowder et al., 2006). There are two Zn^2+^ in the active site of MBLs in subgroups B1 and B3, which can hydrolyze penicillins, cephalosporin and carbapenem antibiotics. The active site of MBLs in the B2 subgroup contains one Zn^2+^, which is dedicated to hydrolyzing carbapenem antibiotics (Rasmussen and Bush, 1997; Garau et al., 2004). Despite only sharing 70.73% amino acid sequence identity with the function-characterized B1 metallo-β-lactamases, WUS-1 had key conserved residues of the subclass B1 enzymes (Palzkill, 2013). The *bla*_WUS-1_ gene exhibits similar drug resistance phenotypes with the other two subclass B1 β-lactamase genes *bla*_MUS-1_ and *bla*_TUS-1_, which all showed resistance to carbapenems and semisynthetic penicillins and could not be inhibited by mechanism-based inhibitors (Mammeri et al., 2002). However, the MIC levels of the cloned *bla*_WUS-1_ gene against the carbapenems (imipenem and meropenem) were higher (increased by 4- and 2-fold, respectively) than those of the *bla*_MUS-1_ or *bla*_TUS-1_ genes (Mammeri et al., 2002), but different from these two enzymes, WUS-1 did not affect MIC levels against cefazolin and cefoxitin, which may be because the activity *in vivo* was simply too low to promote activity *in vitro*. Kinetic analysis showed that WUS-1 presented catalytic behavior similar to that of the closely related class B1 metallo-β-lactamases, which was in accordance with their detected MICs against the β-lactam antimicrobials.

In addition to the novel metallo-β-lactamase gene *bla*_WUS-1_ encoded by the chromosome, this isolate also has two β-lactamase genes, *bla*_MYO-1_ and *bla*_OXA-347,_ carried on a plasmid. No mobile genetic element (MGE) was found in the surrounding areas of the *bla*_WUS-1_ gene, except for a pair of IRs. In contrast to the *bla*_WUS-1_ gene, the two β-lactamase genes together with a number of other resistance genes on the plasmid are all related to mobile genetic elements. All these findings suggest the possible transmission of resistance genes among bacteria of different species or genera.

## Conclusion

In this study, we reported a complete genome sequence of a *Myroides albus* isolate P34 and characterized a novel chromosomally-encoded class B β-lactamase gene, *bla*_WUS-1,_ which confers resistance to some β-lactam antimicrobials, including carbapenems. Carbapenems are used extensively in clinical treatment, and growing resistance to these drugs is a unignorable problem. The production of metalloenzymes plays an important role in the resistance to carbapenems. It remains to be further studied whether these bacteria are reservoirs of such a variety of metalloenzyme genes. Discovering novel resistance genes will be helpful for explaining at least part of their intrinsic resistance to antimicrobials and for finding ways to treat infectious diseases more effectively.

## Data availability statement

The datasets presented in this study can be found in online repositories. The names of the repository/repositories and accession number(s) can be found at: https://www.ncbi.nlm.nih.gov/genbank/, CP102754, CP102755, and UPP01678.1.

## Author contributions

HZ, JL, TX, and QB conceived and designed the experiments. SL, LZ, AL, JZ, YZ, MG, WS, QL, and JXZ performed the experiments. SL CF, JL, and QB contributed to data analysis and interpretation. SL, LZ, TX, and QB contributed to drafting of the manuscript. All authors contributed to the article and approved the submitted version.

## Funding

This study was supported by the Science and Technology Project of Wenzhou City, China (N20210001), Zhejiang Provincial Natural Science Foundation of China (LY19C060002 and LQ17H190001), National Natural Science Foundation of China (81960381 and 81973382), and the Science and Technology Project of Jinhua City, China (2022-2-013).

## Conflict of interest

The authors declare that the research was conducted in the absence of any commercial or financial relationships that could be construed as a potential conflict of interest.

## Publisher’s note

All claims expressed in this article are solely those of the authors and do not necessarily represent those of their affiliated organizations, or those of the publisher, the editors and the reviewers. Any product that may be evaluated in this article, or claim that may be made by its manufacturer, is not guaranteed or endorsed by the publisher.

## Footnotes

^1^ http://www.softberry.com/berry.phtml?topic=bprom&group=programs&subgroup=gfindb

^2^ https://web.expasy.org/protparam/
