# Supplementary material for: Characterization and Identification of a novel chromosome-encoded metallo-β-lactamase WUS-1 in Myroides albus P34
Source: Front Microbiol. 2022 Dec 1;13:1059997. doi: 10.3389/fmicb.2022.1059997 (PMC9751785; doi:10.3389/fmicb.2022.1059997)
Supplement: Supplementary file 1 [file Table_1.DOCX]

**TABLE S1** | Antibiotic detection wavelengths for kinetic studies

| Antibiotics | Wavelength (nm) |
| --- | --- |
| Carbenicillin | 235 |
| Ampicillin | 235 |
| Ticarcillin | 235 |
| Cefoxitin | 260 |
| Cefazolin | 260 |
| Imipenem | 300 |
| Meropenem | 300 |
| Aztreonam | 320 |
| Ceftazidime | 260 |
| Cefepime | 260 |
