# Supplementary material for: Characterization and Identification of a novel chromosome-encoded metallo-β-lactamase WUS-1 in Myroides albus P34
Source: Front Microbiol. 2022 Dec 1;13:1059997. doi: 10.3389/fmicb.2022.1059997 (PMC9751785; doi:10.3389/fmicb.2022.1059997)
Supplement: Supplementary file 2 [file Table_2.DOCX]

**TABLE S2** | General features of the *Myroides albus* P34 genome.

|  | **Chromosome** | **Plasmid** |
| --- | --- | --- |
| Size (bp) | 3,701,500 | 84,474 |
| GC content (%) | 34.18% | 31.07% |
| ORFs | 3,218 | 95 |
| Known proteins | 2,544 | 31 |
| Hypothetical proteins | 674 | 64 |
| Protein coding (%) | 79.05% | 32.6% |
| Average ORF length (bp) | 980 | 759 |
| Average protein length (aa) | 326 | 252 |
| tRNAs | 108 | 0 |
| rRNA operons | (16S-23S-5S)*9 | 0 |
